# Systematic determination of the mosaic structure of bacterial genomes: species backbone versus strain-specific loops

**DOI:** 10.1186/1471-2105-6-171

**Published:** 2005-07-12

**Authors:** H Chiapello, I Bourgait, F Sourivong, G Heuclin, A Gendrault-Jacquemard, M-A Petit, M El Karoui

**Affiliations:** 1 Mathématique, Informatique & Génome, INRA Domaine de Vilvert, 78352 Jouy-en-Josas cedex, France; 2 Unité de Recherches Laitières et Génétique Appliquée, INRA Domaine de Vilvert, 78352 Jouy-en-Josas cedex, France

## Abstract

**Background:**

Public databases now contain multitude of complete bacterial genomes, including several genomes of the same species. The available data offers new opportunities to address questions about bacterial genome evolution, a task that requires reliable fine comparison data of closely related genomes. Recent analyses have shown, using pairwise whole genome alignments, that it is possible to segment bacterial genomes into a common conserved backbone and strain-specific sequences called loops.

**Results:**

Here, we generalize this approach and propose a strategy that allows systematic and non-biased genome segmentation based on multiple genome alignments. Segmentation analyses, as applied to 13 different bacterial species, confirmed the feasibility of our approach to discern the 'mosaic' organization of bacterial genomes. Segmentation results are available through a Web interface permitting functional analysis, extraction and visualization of the backbone/loops structure of documented genomes. To illustrate the potential of this approach, we performed a precise analysis of the mosaic organization of three *E. coli *strains and functional characterization of the loops.

**Conclusion:**

The segmentation results including the backbone/loops structure of 13 bacterial species genomes are new and available for use by the scientific community at the URL: .

## Background

Systematic genome comparisons play an increasingly important role in genome analysis and annotation. There are mainly two kinds of approaches used for whole genome comparisons: whole proteome comparison studies and whole genomic sequence alignment studies. Both approaches are powerful tools to study genome organization and evolution rules with different time scale considerations. These approaches have been employed with success in a recent study comparing the genome of yeast *S. cerevisiae *to three related yeast species genomes [1,2]. Genomewide comparative analysis of the yeast chromosomes has considerably improved gene annotation and has permitted the prediction of new motifs conserved in intergenic regions that act potentially as regulatory elements of gene expression [1].

Whole genome-alignments tools have shown important developments in the last years. It is now possible to align rapidly two or more long genomic DNA sequences with several tools like MultiPipMaker [3], Vista [4], Mummer [5-7] and MGA [8]. Some of them include graphical interfaces to display and browse genome alignments [3,4,7]. Other resources provide precomputed alignments for genome of related species, such as EnteriX or Colibase for enterobacteria [9,10].

Here we focus on whole genomic sequence alignments in the particular case of strains of single bacterial species. Since the publication of a second strain of *Helicobacter pylori *in 1999 [11], sequence data on closely related bacterial genomes has rapidly accumulated in public databases. The availability of complete genome sequences for multiple strains of numerous species opens up new perspectives for studying short term evolutionary processes. For example pairwise alignment of the complete genomes of the enterohemorrhagic *Escherichia coli *0157:H7 strains (Sakaï or EDL 933) with the *E. coli *K-12 laboratory strain, allowed the definition of a 4.1 Mb sequence that was highly conserved between the two strains [12,13]. It was proposed that this common sequence corresponds to the conserved backbone of the *E. coli *chromosome, which is interrupted by numerous DNA segments called strain-specific loops, distributed throughout the backbone [12].

Examination of "mosaic" structures of backbones and loops offers a potential approach to trace the dynamics of genome evolution at the bacterial species level. The backbone, conserved in all aligned genomes of the species, probably corresponds in large part to the common ancestral strain and is the part of the genome under vertical selective pressure. As such, the backbone is also likely to be the most adapted part of the genome, which could be relevant when studying essential functional elements of the cell (such as genes, motifs or signals). Loops differ among strains. Some may correspond to mobile elements, like prophage [14] and insertion sequences [15], and may be associated with strain-specific pathogenicity. However little is known about functional elements associated with small loops.

Up to now, no systematic strategy for backbone/loop segmentation has been proposed for closely related bacterial genomes. The existing studies are either limited to pairwise comparisons or choose a reference genome which is compared to several related genomes. Precomputed alignments are often limited to a subgroup of species and use different softwares and parameters, making results generally non-reproducible or non-comparable.

In this paper we address the problem of defining a strategy to obtain a backbone/loop segmentation of bacterial genomes at the intraspecies level. This approach is based on two recent genome aligners: Mummer3 [7] and MGA [8]. Using a validated benchmark dataset, we developed a simple treatment of alignment results which permits a robust definition of the mosaic structure. Our approach does not take any genome as a reference and has no restriction for the number of genomes to align. We used our method to define this segmentation for 13 bacterial species. Validated backbone/loop segmentation results are stored in the MOSAIC database and are freely accessible through a user friendly Web interface. The backbone/loop segmentation determined using three *E. coli *genomes illustrates important properties of this structure, and indicates that intraspecies segmentation is a useful mean of enhancing bacterial genome annotation.

## Results

**Figure 1:**
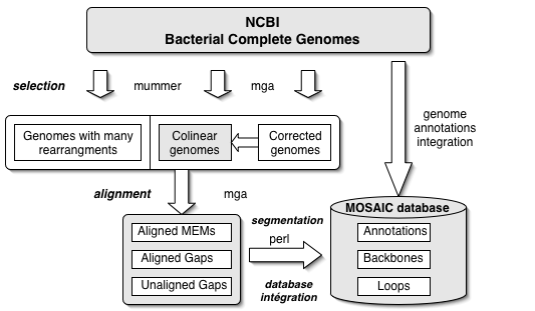
**Flow diagram of bacterial genomes segmentation in MOSAIC. **The bacterial genomes segmentation includes four main steps in MOSAIC: NCBI bacterial genomes selection using Mummer and MGA, processing of genome alignments using MGA, backbone/loops segmentation and database integration using Perl scripts.

The global strategy of genome segmentation and database integration used in this study is outlined Figure 1. A reference set, consisting in a manually verified genome alignment, was used to set appropriate segmentation parameters. Using this strategy, alignments and segmentations were performed systematically for 13 bacterial species, for which at least two genomes have been sequenced. Loop and backbone coordinates were then integrated in the MOSAIC database together with NCBI genome annotations.

### Validation of segmentation parameters

The loop coordinates of the *E. coli *K-12 and O157:H7 Sakai genomes validated by Hayashi *et al*. [16] (see Methods) were used as a basis to define an alignment strategy and to develop a treatment of alignment results adapted to bacterial backbone/loop segmentations. These strains are known to belong to distantly related *E. coli *lineages [17] and their genomes are more distantly related between each other compared with genomes within other species [18]. Parameters allowing a pertinent alignment of such different genomes were therefore expected to produce reliable results for more closely related strains. The K-12/Sakai comparisons were performed using different parameters of the MGA software, and those leading to the best results, as compared to coordinates obtained by Hayashi *et al*., were chosen. This set was used to produce alignments for all species so that results may be compared.

MGA software provides three types of results: matches (anchored MEMs of a minimal given length), aligned gaps (segments between anchored MEMs, shorter than a user-defined size and aligned with ClustalW) and unaligned gaps. Matches were computed using an iterative process on MEM size: MEM of at least 50 bp were used for the first MGA step and MEM of at least 20 bp were computed in the second recursive step. These two kinds of MEM were included into the backbone of the segmented genomes. The gaps were then treated as follows : gaps longer than 3000 bp (unaligned MGA gaps) were considered as loops, and gaps shorter than 3000 bp were aligned with ClustalW. Aligned gaps with more than 76 % identity were considered as backbone, others, as loops. Minimal size of loops and backbone segments was set to 20 nucleotides each. This strategy generated a backbone/loop profile of the K-12/Sakai alignment that differed by around 2400 nt (0,1 %) from that validated by Hayashi *et al*. [16].

### Genome selection for the backbone/loop segmentation

In order to select a subset of genomes for which backbone/loop segmentation makes sense, an analysis using the Mummer package was performed (see Methods). Three categories of results we obtained. The first category includes 33 genomes for which MGA alignments and backbone/loop segmentations are feasible, as they have not been submitted to numerous and important rearrangements. The second category includes genomes that can be aligned after minor correction of their sequences (Reverse complement and Translation operators, see Material & Methods section). This second category concerns 5 genomes (4 species). The last category corresponds to 17 genomes belonging to 8 species: *Neisseria meningitis, Prochlorococcus marinus, Salmonella enterica, Shigella flexneri, Streptococcus pyogenes, Tropheryma whipplei, Xylella fastidiosa *and *Yersinia pestis*. These genomes were excluded because Mummerplot results revealed rearrangements covering a large part of the genome.

### Genome alignments and backbone/loop segmentation

Twenty four genome alignments were generated and treated for backbone/loop segmentation using MGA and our defined set of parameters. These included two quadruple alignments (*E. coli, C. pneumoniae*), four triple alignments (*C. pneumoniae, E. coli, S. aureus, S. pyogenes*) and eigthteen pairwise alignments. For one species, *Buchnera aphidicola*, segmentation results were not exploited due to too low coverage (this value estimates the percentage of total genome length included in the backbone, in this case 40 %, see Discussion).

Validated segmentation results including backbone size, loop size, loop number and genome coverage are described in Table 1. The coverage ranged from 68 % for *E. coli *quadruple alignment to 99 % for *C. pneumoniae *strains. Species comparisons giving high coverage values may be a consequence of to the choice of closely related strains for sequencing, but may also indicate that overall horizontal transfer is less important in some species than in others.

**Table 1:** Segmentation results obtained from MGA alignments and included in the MOSAIC database. For each segmentation result, the first column describes the species and genomes used for segmentation analyses; the number of compared strains is indicated between parentheses. Total loop sizes and loop number of each genome are entered in the same order as strain names, and separated by '+'. Coverage corresponds to the ratio between backbone size and total genome size of a strain; here the mean value for all compared strains is given in percents.

**Compared genomes (numbers of strains)**	**Backbone size (Mb)**	**Cumulative loop size (kb) *[Loop number]***	**Coverage (mean)**
***Agrobacterium tumefaciens***			
C58 Cereon circ X C58 Univ. Wash circ **(2)**	2.09	751*[24]*+751*[25]*	74 %
C58 Cereon lin RC X C58 Univ. Wash lin **(2)**	1.82	252*[13]*+253*[13]*	88 %
***Bacillus anthracis***			
Ames X Ames 'Ancestor' **(2)**	3.93	528*[26]*+528*[24]*	90 %
***Bacillus cereus***			
ATCC14579 X ATCC10987 **(2)**	4.02	1390*[2878]*+1203*[2872]*	76 %
***Chlamydophila pneumoniae***			
AR39 RC+TR X CWL029 X J138 X TW183 **(4)**	1.22	10*[13]*+10*[13]*+7*[11]*+6*[12]*	99%
CWL029 X J138 X TW183 **(3)**	1.21	15*[14]*+11*[12]*+10*[13]*	99 %
CWL029 X J138 **(2)**	1.21	21*[15]*+17*[14]*	99 %
J138 X TW183 **(2)**	1.22	9*[9]*+8*[10]*	99 %
CWL029 X TW183 **(2)**	1.22	13*[6]*+9*[6]*	99 %
AR39 RC+TR X CWL029 **(2)**	1.22	8*[7]*+8*[7]*	99%
***Escherichia coli***			
K-12 X Sakai X EDL933 X CFT073 **(4)**	3.52	1119*[848]*+1978*[830]*+2008*[830]*+1711*[811]*	68 %
K-12 X Sakai X CFT073 **(3)**	3.73	904*[827]*+1763*[795]*+1496*[770]*	73 %
***Helicobacter pylori***			
26695 X J99 **(2)**	1.24	428*[957]*+403*[967]*	75 %
***Listeria monocytogenes***			
EGD X 4b F2365 **(2)**	2.67	270*[644]*+230*[638]*	92 %
***Mycobacterium tuberculosis***			
CDC1551 X H37Rv **(2)**	4.19	217*[164]*+225*[162]*	95 %
***Staphylococcus aureus***			
MW2 X MU50 X N315 **(3)**	2.59	226*[388]*+283*[382]*+220*[388]*	92 %
***Streptococcus agalactiae***			
2603V/R X NEM316 **(2)**	1.88	276*[135]*+327*[132]*	86 %
***Streptococcus pneumoniae***			
R6 X TIGR4 **(2)**	1.91	128*[282]*+250*[294]*	91 %
***Streptococcus pyogenes***			
M1GAS X MGAS315 X MGAS8232 **(3)**	1.62	235*[275]*+283*[282]*+277*[282]*	86 %
M1GAS X MGAS315 **(2)**	1.64	210*[191]*+258*[192]*	88 %
M1GAS X MGAS8232 **(2)**	1.65	206*[225]*+249*[231]*	88 %
***Vibrio vulnificus***			
YJ016 K2 X CMCP6 K2 TR **(2)**	1.63	222*[198]*+210*[199]*	89 %
YJ016 K1 RC X CMCP6 K1 TR **(2)**	2.73	628*[340]*+555*[338]*	82%

The number of loops in a segmented genome appeared to be also highly variable among bacterial species, ranging from 6 (*Chlamydophila pneumoniae *strain CWL29 compared to strain TW183) to 2878 (*Bacillus cereus*, strain ATCC14579 compared to ARCC10987). Results of table 1 revealed two extremes situations. Some species have few very long loops, as *Agrobacterium tumefaciens *(24/25 loops for the circular chromosome, mean length of loops around 28 kilobases). Others (*Bacillus cereus*) contain a large number of short loops (mean length around 400 bases). These differences will need to be further examined in details, in relation with genome annotations.

### Database integration and web interface

**Figure 2:**
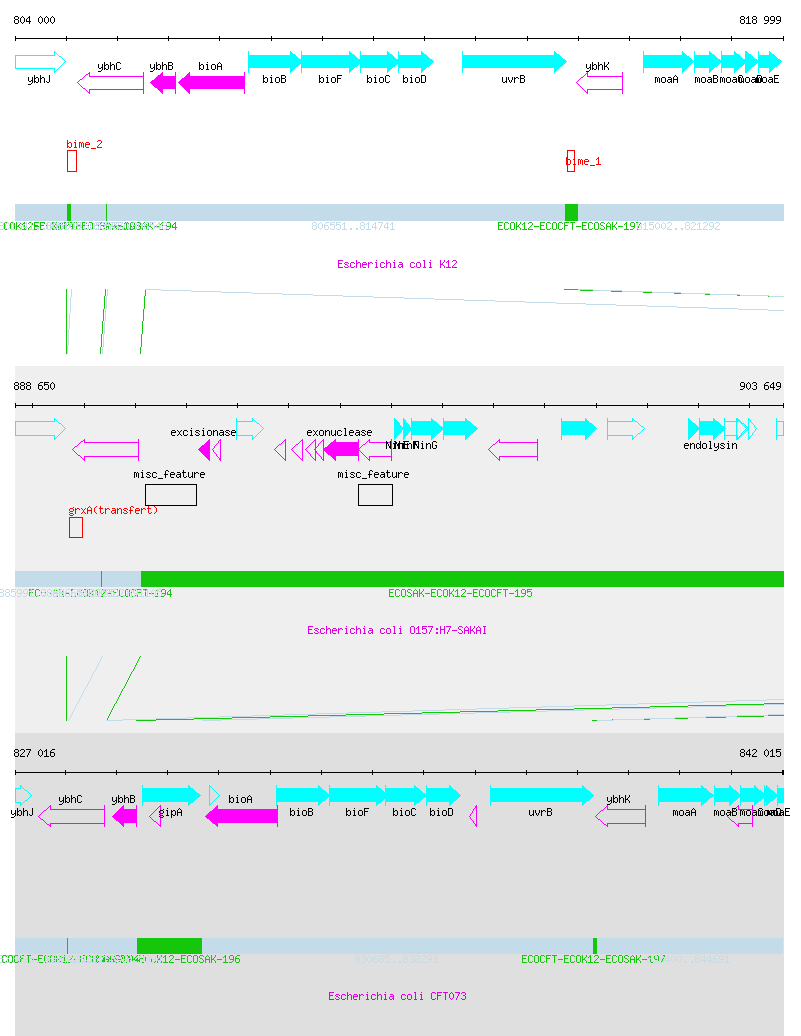
**Graphical visualization of the backbone/loop structure available through the Web interface of Mosaic. **'Physical map' mode of MOSAIC corresponding to the graphical visualization of a 15 kb portion of the *E. coli *K-12, O157:H7 Sakai and CFT073 segmented genomes (data correspond to the comparison of three *E. coli *strains described in results). Genbank annotations are indicated with coloured arrows. Supplementary annotations are indicated as red boxes. Backbone is indicated in grey whereas loops appear in green.

Alignments were integrated into the MOSAIC database and are accessible through the Web interface. Access to the mosaic structure of genomes is made by species selection or gene name selection. For each segmented genome, a local view of the physical map of the segmented genome is available, using MuGeN software [19] (see Figure 2). This graphical visualization of loop and backbone structures is associated with Genbank/NCBI genome annotations. In addition, an overall graphical view of backbone and loops structure is presented using EMBOSS cirdna program. Finally, lists of loop and backbone segments can also be inspected and downloaded according to different criteria like size, genome position or functional characterization.

### In depth analysis of the backbone/loop structure of three *E. coli *genomes

A more precise analysis of the segmentation results from the comparison of *E. coli *strains K-12 [20], O157:H7 Sakai [12] (named Sakai below) and CFT073 [21] (named CFT below) was performed. A 3.73 Mb length backbone (exhibiting more than 97 % identity between the three strains) and three sets of strain-specific loops (of very different total length) were identified. The K-12 genome included 827 K-12 loops (total length 0.9 Mb, 20 % of the K-12 genome), the Sakai genome, 795 Sakai loops (total length 1.8 Mb, 33 % of the Sakai genome) and the CFT genome, 770 CFT loops (total length 1.5 Mb, 29 % of the CFT genome). The differences in total loop sizes are in keeping with the different total genome size of the three strains (K-12: 4.6 Mb ; Sakai: 5.5 Mb ; CFT: 5.2 Mb).

### A large proportion of short loops in the *E. coli *genome

**Figure 3:**
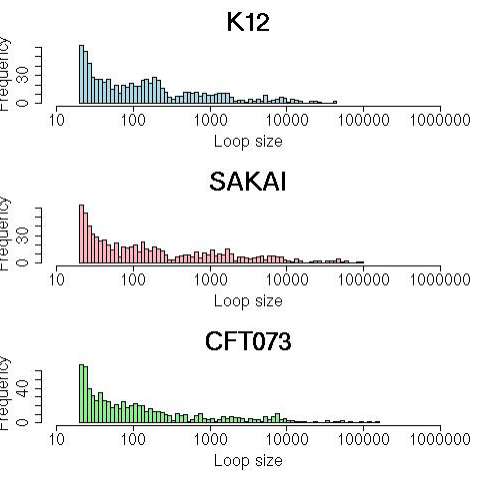
**Distribution of the loop sizes of three E. coli genomes (K-12, SAKAI and CFT073). **Loop sizes range from 20 bp to 40 120 to 151 690 bp. Log10 scale is used on the x-axis.

Basic statistics concerning the size distribution of the three loop sets are described in Table 2 and Figure 3. Results of Table 2 show a remarkable number of short loops for the three species: three quarters of the K-12 loops are shorter than 486 nucleotides (respectively 863 and 314 for Sakai and CFT). The histogram of loop size distribution for the three *E. coli *strains (Figure 3) reveals that the loop population is heterogeneous. Interestingly it appears that the loop populations exhibit roughly the same profile in the three strains, which may comprise three sub-populations: numerous very short loops (length around 100 bp), medium-size loops (length around 1–2 kb) and a few very long loops (length > 10 kb). This may reflect a wide diversity of functional properties conferred by loops: the longer loops probably encode several genes (and correspond for example to bacteriophage or pathogenicity islands). The shorter ones might have regulatory roles or affect gene expression.

**Table 2:** Size distribution of loops (in bp) obtained from segmentation of the *E. coli *genomes K-12, O157:H7 Sakai (SAK) and CFT073 (CFT). Minimal size (Min), Mean size, Maximal size (Max), First Quartile (1st Qu.), Median size, and Third Quartile (3rd Qu.) are shown.

	**K-12 loops**	**SAK loops**	**CFT loops**
**Min**	20	20	20
**Mean**	1093	2217	1942
**Max**	40120	96682	150690
**1st Qu.**	34	32.5	31
**Median**	113	109	77
**3rd Qu.**	486	863	314

### Functional elements associated with backbone and loops

The distribution of functional elements in the backbone/loop structure was analyzed. Functions identified by classical annotations of bacterial genomes i.e. genes, tRNA, rRNA, phages and Insertion Sequences (IS) were first considered. As expected tRNA and rRNA were mainly present in the backbone. One exception concerns a rather large proportion of tRNA present in the Sakai loops (27 %) compared to K-12 (9%) and CFT (13 %) loops. The Sakai strain contains 18 specific tRNAs not present in K-12 [12], Hayashi *et al*. observed that these tRNAs recognize codons which are used with an increased frequency in Sakai loops. Not surprisingly, we observed that phages and IS are quasi-totally included in loops (>98%). The ten longest loops of K-12 correspond systematically to known phages, or phage remnants of the *E. coli *genome.

### One hundred *E. coli *K-12 loops are associated with BIME

To refine functional categorization of smaller loops we examined the correspondence between loops and Bacterial Interpersed Mosaic Element (BIME). BIME are short palindromic repetitive DNA elements found in the genomes of *E. coli *and other enterobacteria [22], and are present exclusively in intergenic regions. BIME are composed of three types of palindromic units (Y, Z1 and Z2). Three sub-families of BIME have been described: BIME-1, which are composed of one Y and one Z1; BIME-2 ("composite" BIME) which contain two to twelve Y and Z2; and a third category ("atypical BIME"), which refers to all other palindromic units associations. BIME sizes range from 140 bp for BIME-1 to several kb for BIME-2 or atypical BIME. BIME are reported to have several functions: mRNA stabilization, transcription termination, translational control and genomic rearrangements [22]. Using the MOSAIC database we identified 100 loops associated with BIME. BIME coordinates were obtained from the "short repeated palindromes in enterobacteria" Web site [23]. They are distributed as follows: in 31 cases, a BIME was present within the loop. In 29 cases, the BIME covered the entire loop region and extended into flanking backbone sequences. In 40 cases, the BIME accounted for more than 50% of the loop length and extended over to one side of the backbone. Results concerning BIME distribution in backbone and loops on the K-12 genome (Table 3) clearly indicate that loops are enriched in BIME elements: in particular 2/3 of the DNA regions associated to BIME are located on the loops. This tendency is particularly striking for BIME-1, for which 71 % of the cumulated length is associated with loops. Interestingly, BIME-2 are quasi equally distributed between backbone and loops. This result is a generalization to all K-12 BIMEs of a result observed in a previous work [24]. PCR analysis of 3 BIME-1 and 3 BIME-2 loci in 51 *E. coli *and *Shigella *isolates showed that BIME-1 are either present or absent among isolates whereas BIME-2 are generally present in the same set of isolates but exhibit a high level of length polymorphism [24]. Figure 2 illustrates two examples of loops associated with BIME-1 and BIME-2, as visible with MuGeN through the MOSAIC Web interface. This association of loops with BIME is a first clue in characterizing the functionality of short loops in *E. coli *strains.

**Table 3:** Distribution of BIME (in percent of length) in backbone and loops regions of the *E. coli *K-12 genome, as determined from the triple K-12, Sakai and CFT073 alignment.

	**K-12**	
	**Backbone**	**Loops**

**BIME**	38 %	62 %
**BIME 1**	29 %	71 %
**BIME 2**	47 %	53 %
**atypical BIME**	37 %	63 %

## Discussion

### Backbone/loop segmentation as a step towers analysis of genome evolution

Studying backbone and loops of bacterial genomes is an efficient way to distinguish the two major modes of evolution acting on bacterial genomes. The backbone may be considered as the part of the genome susceptible to vertical long-term evolution. Backbones are very similar for closely related strains and variability comes mainly from punctual mutations or insertions/deletions of oligonucleotides. The loop population (defined in MOSAIC as variable regions of 21 bp or more) is more heterogeneous : the number of loops and the average loops length varies greatly from one species to another (Table 1). Loops can be viewed as elements issued from short-term evolution processes. One such process is horizontal transfer. For example acquisition/loss of distinct prophage sets seems to be a rapid process, which can be observed between closely related strains [25]. Significantly, for some genomes, phages are the major contributors to loop length [14]. Eleven loops of *E. coli *K-12 are associated with phages and constitute 24 % of the total *E. coli *K-12 loop length. A contrasting example is found in *H. pylori*: this species does not contain prophage, although it contains large loops that may be associated with pathogenicity islands [11]. Our results indicate that medium-size loops (scale of the gene size) are constituted, at least in part, from known variable elements of bacterial genomes like Insertion Sequences. The relatively large number of short loops found in some species (*E. coli*, *B. cereus*) is quite surprising. Such small loops may be due to replication errors ('copy-choice' of DNA polymerase, slippage mechanism), which can generate small insertions or deletions [26] or may correspond to highly polymorphic regions. As opposed to large or medium size loops it is likely that these shorter loops arose from non-horizontal transfer events.

### Consequences of segmentation from multiple alignments

Alignments including more than two genomes generally yield a more robust but smaller backbone than pairwise alignments. This is due to the fact that a larger set of genome variations is taken into account. In the future, about ten or more genomes will be available for some species. One possible consequence is that the backbone length will shrink steadily with new strain genomes. In that case, the backbone may rather be redefined as, for example, the subset of chromosomal regions present in at least half of the strains. Alternatively, the backbone size may decrease but rapidly reach a minimal size, which will be stable even when new strain genomes will be added for alignment.

As a consequence of multiple comparisons, loop populations are greater and more heterogeneous. They include for example elements present in only one genome (which may correspond to acquisition of a very specific characteristic by one strain), elements present in a subset of strains or elements present in all genomes but one (which may correspond to a deletion in one strain). It will be important to systematically classify loops obtained from multiple comparisons in order to facilitate their identification through the MOSAIC interface.

To estimate the importance of loops corresponding to DNA present in the common ancestor but lost in one of the compared strains a preliminary study was performed: all sequences present in the K-12 loops (from the triple alignment) were blasted against the *Salmonella typhimurium *genome (considered as the outgroup), and matching sequences present in the same genome environment were considered as "ancestral loops". Ten loops, corresponding to a total length of 3658 bp, matched this criterium. This suggests that only a minor subset of the loops correspond to deletions that occurred in either *E. coli *Sakai or CFT genomes.

### Backbone/loops segmentation for divergent or rearranged genomes

Some genomes of species like *Buchnera aphidicola *were too divergent to be segmented with our procedure. In fact, these genomes are clearly atypical in terms of evolutionary distance within a species: despite complete colinearity of their genomes, *B. aphidicola *Sg and Ap genomes display a high degree of divergence at the nucleotide level, making them as different as *E. coli/S. typhimurium *genomes [27]. Comparison of Sg and Ap genomes is thus almost the same situation as comparing different species, but would be possible by adapting the alignment parameters. This raises the question of bacterial species definition: the evolutionary distances within a species and between species are very heterogeneous. For example, it has recently been confirmed that *Shigella *is phylogenetically indistinguishable from *E. coli *[28]. Our method will also be easily extended to bacterial species where numerous chromosomal rearrangements have occurred, using recently developed genome aligners such as MAUVE [29]. Intra-species comparison of divergent and/or rearranged genomes will open the way to segmentation of genomes from different, but closely related species.

### A new category of genome annotation

To our knowledge, this work is the first study allowing systematic mosaic genome segmentation of all available strains (ranging from two to four) in 13 bacterial species. Examination of the backbone of a bacterial species should greatly facilitate refinement of gene annotation and prediction of conserved sites with potential regulatory roles. Examination of the gene content in loops is important for identification of putative horizontally transferred genes. Genes adapted to specific ecological environments or involved in pathogenicity of a specific strain should also be found in the strain-specific loops. Indeed, the ASAP database (A Systematic Annotation Package for community analysis of genomes) [30] recently added the features type 'island' and 'conserved_segments' in order to provide lists of regions that are specific or common to the two *E. coli *K-12 and O157:H7 genomes.

## Conclusion

Genome aligners were used to build a robust strategy for bacterial genome segmentation. Backbone/loops structures were systematically determined for 38 bacterial genomes. The MOSAIC resource makes it easy to visualize, annotate, and analyse loops and backbone segments of these genomes. First analyses reveal a surprising diversity in the number of loops from one species to another. In addition some species accumulate a large number of short loops, unsuspected previously.

## Methods

### Species selection

Complete bacterial genomes were downloaded from the NCBI microbial genome database: , version of 06/24/2004. Twenty one species (55 genomes) for which genome sequences of at least two different strains are available were selected for analysis: *Agrobacterium tumefaciens, Bacillus anthracis, Bacillus cereus, Buchnera aphidicola, Chlamydophila pneumoniae, Escherichia coli, Helicobacter pylori, Listeria monocytogenes, Mycobacterium tuberculosis, Neisseria meningitidis, Prochlorococcus marinus, Salmonella enterica, Shigella flexneri, Staphylococcus aureus, Streptococcus agalactiae, Streptococcus pneumoniae, Streptococcus pyogenes, Tropheryma whipplei, Vibrio vulnificus, Xylella fastidiosa*, and *Yersinia pestis *[see Additional file 1].

### Segmentation strategy

Backbone/loop segmentations were determined using a simple procedure based on Mummer3 [7] and MGA [8] results (see figure 1).

### Selection of genomes without rearrangement

In the first step, the subset of genomes for which it is possible to define a reliable backbone was identified using mummer and mummerplot scripts of the Mummer3 package. First, all Maximal Exact Matches (MEM, not necessarily unique) of at least 20 bp in both forward and reverse strands of the compared genomes were computed using the mummer program. Visualization of results between each pair of sequences was then performed using the mummerplot program. This graphical visualization was used to decide whether a common backbone could be defined for the considered genomes. In several cases, this step led us to adjust one of the genomes before the segmentation step. Two operators were defined: the reverse complement operator, named RC, and the translation operator, named TRx, where x indicates that bases from position 1 to x were transferred to the end of the genome. This number x of bases shifted to the end of the genome was determined by the position of the first aligned MEM detected by MGA between the two genomes. These operators allowed us to assign the same strand and the same start position to all compared genomes. They were applied to a subset of genomes before alignment with MGA software. Genomes where rearrangements covering more than half of the total length were detected by mummerplot and excluded at this step. They can not be handled properly by MGA and would therefore lead to inaccurate segmentation.

### Backbone/loop segmentation

The second step was to use the MGA software to perform whole genome alignments on the subset of selected genomes and to define backbone and loops. MGA is a powerful multiple genome aligner which presents two major advantages. First, it performs simultaneous multiple alignments based on MEM (Maximal Exact Matches present in all aligned genomes) selection, without considering any genome as the reference. Second, a consistent and robust backbone for the aligned genomes can be defined using its MEM anchoring algorithm followed by treatment of gaps (i.e. regions between the anchored MEM). Parameters used in MGA were adjusted by comparison with a manually curated reference set of loops of two *E. coli *strains: K-12 and O157:H7 Sakai [16]. After Mummer 1 alignment, backbone/loop junctions were extracted and systematically aligned using the fasta3 algorithm. Each alignment was checked by eye inspection and in many cases, the backbone sequence was extended by a few to several base pair [Pr. T. Hayashi, personal communication]. Further analysis using whole genome PCR scanning confirmed that the loops longer than 500 pb are indeed variable elements [31]. A simple treatment of MGA alignment results was developed to define the boundaries of loops and to enhance their concordance with this manually determined pairwise reference dataset [see 'Results' section, 'Validation of segmentation parameters' subsection]

### Coverage calculation and database integration

Results of MGA alignments were generated in XML format. Backbone/loop segmentations were processed with a Perl script using the SAX module for XML parsing. For each aligned genome, backbone and loop coordinates were computed and coverage (length of the backbone divided by total length of the genome) was calculated. Results were then integrated into the MOSAIC relational database. The database was implemented using the PostgreSQL relational database system. The MOSAIC relational model is generic and not dedicated to any alignment tool or genome species. The Web interface was also written in Perl language using standard modules for database access (DBI module for DataBase Interface) and dynamic pages (CGI module for Common Gateway Interface). Different graphical visualizations of the backbone/loop structure were developed using the MuGeN software [19] and the cirdna program which is part of the EMBOSS package [32].

## Authors' contributions

H. Chiapello performed segmentation results, conceived the MOSAIC application and drafted the manuscript. I. Bourgait, and A. Gendrault-Jacquemard participated in the database design and segmentation results integration. G. Heuclin performed loop analysis. F. Sourivong built the Web interface. M-A. Petit participated in data analysis and helped to draft the manuscript with M. El Karoui who supervised the study.

## Supplementary Material

Additional File 1 The 55 bacterial genomes for which at least two strains have been sequenced. For each species and each strain, NCBI accession number and genome length are indicated. The 'MGA aligt.' column indicates if the genomes have been included in an MGA alignment. Genomes corrections are indicated in the 'Correction column' as follows : '-', no correction, 'RC', Reverse Complement strand, and 'TR+x' means that segment in position 1 to x of the genome has been shifted at the end of the genome. A brief comment is given for genomes excluded from MGA alignments. Click here for file
