# Supplementary material for: Systematic determination of the mosaic structure of bacterial genomes: species backbone versus strain-specific loops
Source: BMC Bioinformatics. 2005 Jul 12;6:171. doi: 10.1186/1471-2105-6-171 (PMC1187871; doi:10.1186/1471-2105-6-171)
Supplement: Additional File 1 — The 55 bacterial genomes for which at least two strains have been sequenced. For each species and each strain, NCBI accession number and genome length are indicated. The 'MGA aligt.' column indicates if the genomes have been included in an MGA alignment. Genomes corrections are indicated in the 'Correction column' as follows : '-', no correction, 'RC', Reverse Complement strand, and 'TR+x' means that segment in position 1 to x of the genome has been shifted at the end of the genome. A brief comment is given for genomes excluded from MGA alignments. [file 1471-2105-6-171-S1.doc]

**Supplementary material**

***Table 1*** *: The 56 bacterial genomes for which at least two strains have been sequenced. For each species and each strain, NCBI accession number and genome length are indicated. The 'MGA aligt.' column indicates is the genomes has been included in an MGA alignment. Genomes corrections whin have been operated are indicated in the 'Correction column' as follows : '-' means no correction, 'RC' means 'Reverse Complement' strand and TR+x means that segment in position 1 to x of the genome has been shifted at the end of the genome. For genome which have been excluded of MGA alignments, a brief comment is given.*

| **Species** | **Strain** | **Accession** | **Length** | **MGA aligt** | **Correction** | **Comment** |
| --- | --- | --- | --- | --- | --- | --- |
| *Agrobacterium tumefaciens* | C58 Cereon circ. | NC_003062 | 2841581 | yes | - | - |
| *Agrobacterium tumefaciens* | C58 Cereon lin. | NC_003063 | 2074782 | yes | RC | - |
| *Agrobacterium tumefaciens* | C58 Univ. Wash. circ. | NC_003304 | 2841490 | yes | - | - |
| *Agrobacterium tumefaciens* | C58 Univ. Wash. lin. | NC_003305 | 2075560 | yes | - | - |
| *Bacillus anthracis* | str. A2012 | NC_003995 | 5093554 | yes | TR+567824 | - |
| *Bacillus anthracis* | str. Ames | NC_003997 | 5227293 | yes | - | - |
| *Bacillus anthracis* | str. 'Ames Ancestor' | NC_007530 | 5227419 | yes | - | - |
| *Bacillus cereus* | ATCC 10987 | NC_003909 | 5224283 | yes | - | - |
| *Bacillus cereus* | ATCC 14579 | NC_004722 | 5411809 | yes | - | - |
| *Buchnera aphidicola* | str. APS | NC_002528 | 640681 | yes | - | Divergent genomes |
| *Buchnera aphidicola* | str. Sg | NC_004061 | 641454 | yes | - | Divergent genomes |
| *Chlamydophila pneumoniae* | AR39 | NC_002179 | 1229858 | yes | RC+TR388657 | - |
| *Chlamydophila pneumoniae* | CWL029 | NC_000922 | 1230230 | yes | - | - |
| *Chlamydophila pneumoniae* | J138 | NC_002491 | 1226565 | yes | - | - |
| *Chlamydophila pneumoniae* | TW-183 | NC_005043 | 1225935 | yes | - | - |
| *Escherichia coli* | K12 | NC_000913 | 4639221 | yes | - | - |
| *Escherichia coli* | O157:H7 EDL933 | NC_002655 | 5528445 | yes | - | - |
| *Escherichia coli* | O157:H7-SAKAI | NC_002695 | 5498450 | yes | - | - |
| *Escherichia coli* | CFT073 | NC_004431 | 5231428 | yes | - | - |
| *Helicobacter pylori* | 26695 | NC_000915 | 1667867 | yes | - | - |
| *Helicobacter pylori* | J99 | NC_000921 | 1643831 | yes | - | - |
| *Listeria monocytogenes* | str. 4b F2365 | NC_002973 | 2905310 | yes | - | - |
| *Listeria monocytogenes* | EGD-e | NC_003210 | 2944528 | yes | - | - |
| *Mycobacterium tuberculosis* | H37Rv | NC_000962 | 4411529 | yes | - | - |
| *Mycobacterium tuberculosis* | CDC1551 | NC_002755 | 4403837 | yes | - | - |
| *Neisseria meningitis* | MC58 | NC_003112 | 2272351 | no | - | Rearrangments |
| *Neisseria meningitis* | Z2491 | NC_003116 | 2184406 | no | - | Rearrangments |
| *Prochlorococcus marinus* | CCMP1375 | NC_005042 | 1751080 | no | - | Rearrangments |
| *Prochlorococcus marinus* | MIT9313 | NC_005071 | 2410873 | no | - | Rearrangments |
| *Prochlorococcus marinus* | CCMP1378 | NC_005072 | 1657990 | no | - | Rearrangments |
| *Salmonella enterica* | Typhi Ty2 | NC_004631 | 4791961 | no | - | Inversion |
| *Salmonella enterica* | Typhi CT18 | NC_003198 | 4809037 | no | - | Inversion |
| *Shigella flexneri* | 301 | NC_004337 | 4607203 | yes | - | - |
| *Shigella flexneri* | 2457T | NC_004741 | 4599354 | yes | - | - |
| *Staphylococcus aureus* | N315 | NC_002745 | 2814816 | yes | - | - |
| *Staphylococcus aureus* | Mu50 | NC_002758 | 2878040 | yes | - | - |
| *Staphylococcus aureus* | Mw2 | NC_003923 | 2820462 | yes | - | - |
| *Streptococcus agalactiae* | 2603V/R | NC_004116 | 2160267 | yes | - | - |
| *Streptococcus agalactiae* | NEM316 | NC_004368 | 2211485 | yes | - | - |
| *Streptococcus pneumoniae* | TIGR4 | NC_003028 | 2160837 | yes | - | - |
| *Streptococcus pneumoniae* | R6 | NC_003098 | 2038615 | yes | - | - |
| *Streptococcus pyogenes* | M1 GAS | NC_002737 | 1852441 | yes | - | *-* |
| *Streptococcus pyogenes* | MGAS315 | NC_004070 | 1900521 | yes | - | *-* |
| *Streptococcus pyogenes* | MGAS8232 | NC_003485 | 1895017 | no | - | - |
| *Streptococcus pyogenes* | SSI-1 | NC_004606 | 1894275 | no | - | Rearrangments |
| *Tropheryma whipplei* | TW08/27 | NC_004551 | 925938 | no | - | Inversion + repeats |
| *Tropheryma whipplei* | Twist | NC_004572 | 927303 | no | - | Inversion + repeats |
| *Vibrio vulnificus* | CMCP6 chr. I | NC_004459 | 1844853 | yes | TR1012685 | - |
| *Vibrio vulnificus* | YJ016 chr. I | NC_005139 | 1857073 | yes | RC | - |
| *Vibrio vulnificus* | CMCP6 chr. II | NC_004460 | 1844853 | yes | - | - |
| *Vibrio vulnificus* | YJ016 chr. II | NC_005140 | 1857073 | yes | TR578956 | - |
| *Xylella fastidiosa* | 9a5c | NC_002488 | 2679306 | no | - | Rearrangments |
| *Xylella fastidiosa* | Temecula1 | NC_004556 | 1544903 | no | - | Rearrangments |
| *Yersinia pestis* | CO92 | NC_003143 | 4653728 | no | - | Rearrangments |
| *Yersinia pestis* | KIM | NC_004088 | 4600755 | no | - | Rearrangments |
| *Yersinia pestis* | 910001 | NC_005810 | 4595065 | no | - | Rearrangments |
